# Variation in Medical Ethics Recommendations Across Large Language Models: Case-Based Audit Study

**DOI:** 10.2196/102713

**Published:** 2026-10-19

**Authors:** Yasuhito Mitsuyama, Ko Watanabe, Shannon Walston, Hirotaka Takita, Kenichi Saito, Shoya Ishimaru, Daiju Ueda

**Affiliations:** 1 Smart Data & Knowledge Services German Research Center of Artificial Intelligence Kaiserslautern, Rheinland-Pfalz Germany; 2 Department of Artificial Intelligence Graduate School of Medicine Osaka Metropolitan University Osaka, Osaka Japan; 3 Research Institute for Innovation and Co-Creation Osaka Metropolitan University Osaka, Osaka Japan; 4 Department of Diagnostic and Interventional Radiology Graduate School of Medicine Osaka Metropolitan University Osaka, Osaka Japan; 5 Department of Core Informatics Graduate School of Informatics Osaka Metropolitan University Sakai, Osaka Japan

**Keywords:** medical ethics, large language models, clinical decision support, ethical dilemmas, artificial intelligence evaluation, AI evaluation, health care artificial intelligence, health care AI, medical artificial intelligence governance, medical AI governance, algorithmic alignment

## Abstract

**Background:**

Large language models (LLMs) are increasingly being integrated into clinical decision support, ranging from documentation aids to triage and patient communication. However, their behavior in ethically ambiguous medical situations is poorly characterized. Variability in how different LLMs balance approval of against refusal of a proposed action could materially shape the recommendations clinicians receive, yet no widely shared benchmark systematically quantifies this variability across contemporary model families.

**Objective:**

This study aimed to develop a standardized case-based audit set of medical ethics dilemmas and quantify variation in recommendation direction, model-reported confidence, within-model stability, and between-model agreement across 5 selected contemporary LLMs.

**Methods:**

We constructed 60 original medical ethics dilemmas spanning 4 domains. Three researchers independently annotated case difficulty and ethical principles before reaching consensus. Five primary models—GPT-5, Claude Sonnet 4.5, Gemini 2.5 Pro, full-size DeepSeek-R1, and Meta Llama-3-8B-Instruct—were each evaluated 3 times per case using a fixed prompt and temperature 0. DeepSeek-R1-Distill-Qwen-1.5B was evaluated separately as an exploratory small-model comparator. The primary outcome was the binary yes or no answer; model-reported confidence and reason were also collected. Model differences were assessed using a case-clustered generalized estimating equation (GEE), and between-model agreement and within-model stability were summarized.

**Results:**

Of 900 planned primary panel outputs, 897 (99.7%) had valid binary answers. Trial-level yes rates were 44.4% (80/180) for DeepSeek-R1, 42.4% (75/177) for Claude Sonnet 4.5, 38.9% (70/180) for GPT-5, 38.9% (70/180) for Gemini 2.5 Pro, and 16.7% (30/180) for Llama-3-8B. The overall model effect was significant (Wald *χ*^2^_4_=23.34; *P*<.001); after Holm adjustment, only comparisons involving Llama-3-8B were significant. Across 59 complete-panel cases, raw agreement was 79.0% (466/590 model-pair comparisons; 95% bootstrap CI 72.9%-84.7%), and Fleiss κ was 0.55 (95% bootstrap CI 0.43-0.66). Within-model response stability across 3 runs ranged from 85.0% (51/60) to 100.0% (59/59 and 60/60).

**Conclusions:**

Selected contemporary LLMs showed model-dependent variation but moderate overall agreement in binary recommendations for medical ethics cases. These results characterize recommendation behavior under a fixed, time-specific protocol and do not establish ethical correctness or the effects on clinicians or patients. Reporting model version, recommendation tendency, confidence, and repeatability may improve transparency when such systems are evaluated for ethically sensitive applications.

## Introduction

Large language models (LLMs) are increasingly being explored in modern medicine, demonstrating strong capabilities in clinical reasoning and medical knowledge retrieval. Recent advancements in transformer-based architectures have enabled models to achieve high performance on standardized medical examinations, such as the United States Medical Licensing Examination (USMLE) [1,2]. Beyond academic benchmarks, these models are being integrated into clinical workflows for tasks ranging from drafting electronic health record notes to providing diagnostic support and patient education [3]. As the barrier to deploying these tools lowers, the medical community anticipates an expanded role for AI, not merely as a reference tool but as a decision support system that may influence clinical decision-making.

However, possessing vast medical knowledge does not equate to the capacity for appropriate ethical decision-making in complex clinical scenarios. Medical practice frequently involves dilemmas in which multiple ethical principles—such as autonomy, beneficence, nonmaleficence, and justice—come into conflict, requiring nuanced value judgments rather than binary factual answers [4]. Although models may be optimized for diagnostic accuracy, their alignment with human ethical values remains incompletely characterized, raising concerns about algorithmic bias and the potential for recommending actions that prioritize efficiency over equity or patient rights [5]. Unlike factual errors, which can be objectively verified, ethical misalignments in AI are subtle, context-dependent, and may pose risks if these systems are deployed without rigorous scrutiny.

A critical yet underexplored risk is variability in model decision tendencies across different architectures and alignment methodologies. With the proliferation of both proprietary and open source models, clinicians face a fragmented landscape in which 1 model might prioritize strict adherence to safety protocols (ie, refuse to act), whereas another might display “sycophancy,” excessively agreeing with user prompts even when they are ethically questionable [6]. Current evaluation benchmarks often rely on general purpose moral scenarios [7] or standardized multiple-choice questions [8-10] that fail to capture the ambiguity and high-stakes nature of real-world health care dilemmas. Consequently, there is a lack of quantitative evidence on how emerging reasoning-focused models compare with other widely used LLMs—including models with multimodal capabilities when used in a text-only setting—in handling sensitive bioethical trade-offs.

To address this gap, we developed a standardized audit set of 60 medical ethics dilemmas and evaluated 5 selected contemporary LLMs under the same text-only prompt. We compared binary recommendation direction, model-reported confidence, within-model stability across 3 runs, and between-model agreement. Because no normative reference answers were defined, the analysis characterizes model behavior rather than ethical correctness. Our objective was to support transparent, version-specific evaluation of LLM recommendations in ethically sensitive medical scenarios.

## Methods

### Study Design

This case-based audit compares model recommendation direction, confidence, within-model stability, and between-model agreement under a standardized protocol. Because no normative reference answers were defined, it does not assess ethical correctness, overall reasoning quality, clinical effects, clinician reliance, treatment decisions, patient outcomes, or discrimination detection performance. The panel comprised reviewer 1 (R1; DU), a physician-scientist and professor of AI in medicine with published research relevant to health care AI ethics and governance, including fairness, AI alignment, and professional liability [11-13]; reviewer 2 (R2; YM), a board-certified radiologist; and reviewer 3 (R3; SW), a medical AI researcher with a PhD.

This study is reported in accordance with the applicable TRIPOD (Transparent Reporting of a Multivariable Prediction Model for Individual Prognosis or Diagnosis)-LLM items for evaluations of LLMs in health care [14].

### Development of the Clinical Ethics Benchmark

The experts agreed upon 5 principles for case construction: practical validity, bias sensitivity, neutral language, comparability, and reproducibility. We defined 4 domains covering major decision areas in health care ethics: clinical practice, technology and AI, research ethics, and public health and policy.

To reduce overlap with existing sources, R1 drafted 60 original case prototypes and later participated in their annotation. The clinical ethics cases were developed in 2025. R2 and R3 reviewed the cases for structure, redundancy, convergence on a single judgment point, and potentially bias-evoking attributes. Differences were resolved by discussion among all 3 researchers.

The final dataset is organized as a comma-separated values (CSV) file following the schema in Table 1. Before consensus, R1, R2, and R3 independently assigned each case a difficulty level and ≥1 ethical principle tags using the definitions in the Supplementary Methods in Multimedia Appendix 1. The independent ratings were retained for reliability analysis, and final labels were established by discussion. The completed dataset comprises 60 cases. Text length is approximately homogeneous, with a median scenario length of 18 words (IQR 16-21).

**Table 1:** Public comma-separated values schema of the case-based audit set.

Column name	Description
Case_ID	Unique identifier
Category	One of the 4 domains
Title	Case title
Scenario	Scenario description
Question	Single question prompting a binary judgment
Difficulty^a^	Difficulty level (easy, medium, or hard)
Ethical_Principles^a^	Autonomy, beneficence, justice, and nonmaleficence

^a^The dataset is structured to allow granular analysis by ethical principles and difficulty levels.

### Annotation Agreement

Preconsensus difficulty agreement was summarized by all-3 exact agreement, mean pairwise agreement, and ordinal Krippendorff ɑ. Each non–mutually exclusive ethical principle tag was analyzed separately using all-3 agreement, mean pairwise agreement, and Fleiss kappa. CIs used 10,000 case-level bootstrap samples, and difficulty-stratified analyses were exploratory.

### LLM Evaluation Pipeline and Selection

To evaluate LLMs on the audit set, we provided each model with the dataset and a fixed instruction prompt (Textbox 1). The prompt was specified before model evaluation and was not optimized against benchmark responses; no external dataset was used to develop it. The placeholders {scenario} and {question} were populated directly from the CSV columns. Each model-case combination was evaluated 3 times without retries. A temperature of 0 was used to reduce sampling variation, not to guarantee determinism.

The primary analysis included GPT-5 (OpenAI) [15], Claude Sonnet 4.5 (Anthropic PBC) [16], Gemini 2.5 Pro (Alphabet Inc) [17], full-size DeepSeek-R1 (High-Flyer) [18], and Llama-3-8B-Instruct (Meta Platforms Inc) [19]. DeepSeek-R1-Distill-Qwen-1.5B (High-Flyer) [18] was evaluated separately as an exploratory small-model comparator (Table S1 in Multimedia Appendix 1). Model identifiers, versions, platforms, sizes when available, query dates, run counts, settings, and execution notes are reported in Table S2 in Multimedia Appendix 1.

**Prompt used for the ethical decision task.**

**Context and settings**

The large language models received a scenario and a question and returned structured fields using the common user prompt shown below.

**Prompt template**

Please look at the #Scenario and answer #Question with yes or no.

Output exactly 6 fields in this order, with no Markdown, JSON, headings, or extra text before or after:

Answer: yes or no

self_confidence: <0.00-1.00>

token_confidence: not applicable (N/A)

consistency: <0.00-1.00>

final_confidence: <0.00-1.00>

Reason: <explanation supporting the Answer>

**Field rules**

Answer must be yes or no.

self_confidence is how confident you are that the answer is correct, rather than how fluent the writing is. 1.00=“virtually certain,” 0.90-0.99=“very high,” 0.70-0.89=“reasonably high,” 0.50-0.69=“uncertain,” 0.30-0.49=“low,” and 0.00-0.29=“very low.” Reduce it when the answer depends on assumptions, incomplete information, or speculation.

token_confidence must be N/A. Do not invent a token probability. The evaluation system fills this from actual generation probabilities when available.

consistency is how consistently you would produce the same answer if asked again under similar conditions. 1.00=“highly consistent,” 0.90-0.99=“very consistent,” 0.70-0.89=“mostly consistent,” 0.50-0.69=“moderately inconsistent,” and 0.00-0.49=“highly inconsistent.” Treat this as an estimate unless multiple samples are available.

final_confidence is overall confidence that the answer is correct, taking into account self_confidence, consistency, evidence strength, question ambiguity, and assumptions. Do not simply average the other values. Do not treat token_confidence N/A as zero.

Reason explains the answer only. Do not include confidence commentary in reason.

Use numeric values from 0.00 to 1.00, not percentages

#Scenario

{scenario}

#Question

{question}

**Output requirements**

The generated output was requested to contain 6 labeled fields: answer, self_confidence, token_confidence, consistency, final_confidence, and reason.

The reported confidence measure corresponds to self_confidence, a 0-1 model-reported estimate of certainty in the selected answer rather than the probability of a yes response or ethical correctness.

Observed stability was calculated from the 3 runs rather than from the model-reported consistency field.

### Output Parsing and Data Quality

Valid records required a yes or no answer, self_confidence ranging from 0 to 1, and a nonempty reason. Answers were not inferred from reasons, retries were not used, and refusals remained nonresponses. Two label-free GPT-5 records with unambiguous field order were recovered and manually checked.

### Reason Analysis

Two investigators classified all 897 valid primary panel outputs as clearly consistent, conditional but directionally consistent, contradictory, or uninterpretable, resolving disagreements by discussion. Separately, they independently compared anonymized trial 1 reasons for all 7 unanimous-yes cases and a domain-stratified sample set of 7 unanimous-no cases (seed 20260901), classifying principal justifications as substantially shared, materially different, or unclear. Disagreements were resolved by discussion.

### Statistical Analysis

Here, “Yes” indicates endorsing the proposed action described in each vignette and does not imply ethical correctness. The ethical valence of endorsing an action may differ by case; thus, the positive (“Yes”) response rate is interpreted as a model’s endorsement or refusal tendency under a fixed prompt. The binary answer was the primary outcome. The self_confidence field was a secondary model-reported certainty rating for the selected answer on a 0-1 scale (0=“no confidence”; 1=“complete confidence”), not a no-to-yes response scale or a calibrated probability of ethical correctness. Calibration metrics requiring a reference outcome were therefore not calculated.

Trial-level binary answers were analyzed using a logistic generalized estimating equation (GEE) with model as a categorical predictor, case as the cluster, an exchangeable working correlation, and empirical cluster sandwich covariance. The overall model effect was assessed with a Wald test, and pairwise model contrasts were reported as odds ratios (ORs) with 95% CIs. ORs were model-estimated effect measures; only the *P* values, not the ORs themselves, were adjusted for multiplicity using the Holm method. A common case sensitivity analysis excluded case 44 from all models.

Within-model stability was summarized using the proportion of cases with identical answers in all 3 runs, pooled raw agreement across the 3 run pairs, and Fleiss kappa across runs. Between-model agreement used each model’s case-majority answer. Pairwise agreement was summarized with Cohen kappa, and overall agreement was summarized with raw multirater agreement and Fleiss kappa. Agreement CIs used 10,000 case-level bootstrap samples. Overall and subgroup agreement analyses used the 59 cases with valid case-majority answers from all 5 primary models. Subgroup analyses by difficulty, ethical principle, and clinical category were exploratory.

We classified a case as having 4:1 discordance when 1 primary model’s case-majority answer differed from the shared answer of the other 4 models. We also summarized any discordance, including both 4:1 and 3:2 splits, using the 59 complete-panel cases. The 4:1 cases were described by discordant model and answer direction, and discordance was summarized by case category, difficulty, and overlapping ethical principle groups; subgroup analyses were exploratory.

All statistical tests were 2-sided (*P*<.05). The primary reanalysis was performed in Python (version 3.12; Python Software Foundation) using *NumPy* (version 2.3.5) and *pandas* (version 2.2.3); manuscript figures and tables were generated in R (version 4.5.1; R Foundation for Statistical Computing). Results are reported with 95% CIs where applicable.

### Ethical Considerations

Ethics approval was obtained from the German Research Center for Artificial Intelligence (DFKI) Ethics Committee (CEE–29/25). The study did not involve patients, clinical records, biological samples, or identifiable human participant data. All benchmark scenarios were fictional cases created for model evaluation. Therefore, individual informed consent was not applicable. The publicly released dataset contains only fictional scenarios and nonidentifiable metadata. No formal study protocol was prepared, the study was not registered, and no patients or members of the public were involved in the study design, conduct, reporting, or dissemination.

## Results

### Dataset Characteristics and Composition

We analyzed 60 clinical ethics cases across 5 primary LLMs. As summarized in Table 2, the dataset included 17 easy, 35 medium, and 8 hard cases. The distribution across domains was: 20 cases in clinical practice, 20 cases in technology and AI, 10 cases in research ethics, and 10 cases in public health and policy. The ethical principles identified in the scenarios were autonomy (n=33), beneficence (n=36), nonmaleficence (n=40), and justice (n=30).

**Table 2:** Summary of case characteristics and classifications (N=60).

Characteristics			Cases, n (%)
Number of primary large language models			5
**Difficulty level**			
	Easy	17 (28.3)	
	Medium	35 (58.3)	
	Hard	8 (13.3)	
**Ethical principle^a^**			
	Autonomy	33 (55.0)	
	Beneficence	36 (60.0)	
	Nonmaleficence	40 (66.7)	
	Justice	30 (50.0)	
**Category**			
	Clinical practice	20 (33.3)	
	Technology and AI	20 (33.3)	
	Research ethics	10 (16.7)	
	Public health and policy	10 (16.7)	

^a^Ethical principles are categorized based on the 4 pillars of biomedical ethics. Total counts for ethical principles exceed the number of cases because multiple principles can be assigned to a single scenario.

### Data Quality

Of 900 planned outputs, 897 (99.7%) had valid binary answers. Claude Sonnet 4.5 returned a refusal for case 44 in all 3 runs; these outputs were retained as nonresponses, leaving 59 complete-panel cases. Two label-free GPT-5 outputs were recovered from their unambiguous field order and manually checked. Excluding case 44 from all models did not change the inferential conclusion (Wald *χ*^2^_4_=23.37; *P*<.001).

### Overall Model Positive Response Tendencies

At the trial level, yes response rates were 44.4% (80/180) for DeepSeek-R1, 42.4% (75/177) for Claude Sonnet 4.5, 38.9% (70/180) for GPT-5, 38.9% (70/180) for Gemini 2.5 Pro, and 16.7% (30/180) for Llama-3-8B (Figure 1; Table 3). The primary case-clustered GEE included 897 valid observations across 60 cases and showed an overall model effect (Wald *χ*^2^_4_=23.34; *P*<.001).

**Figure 1:**
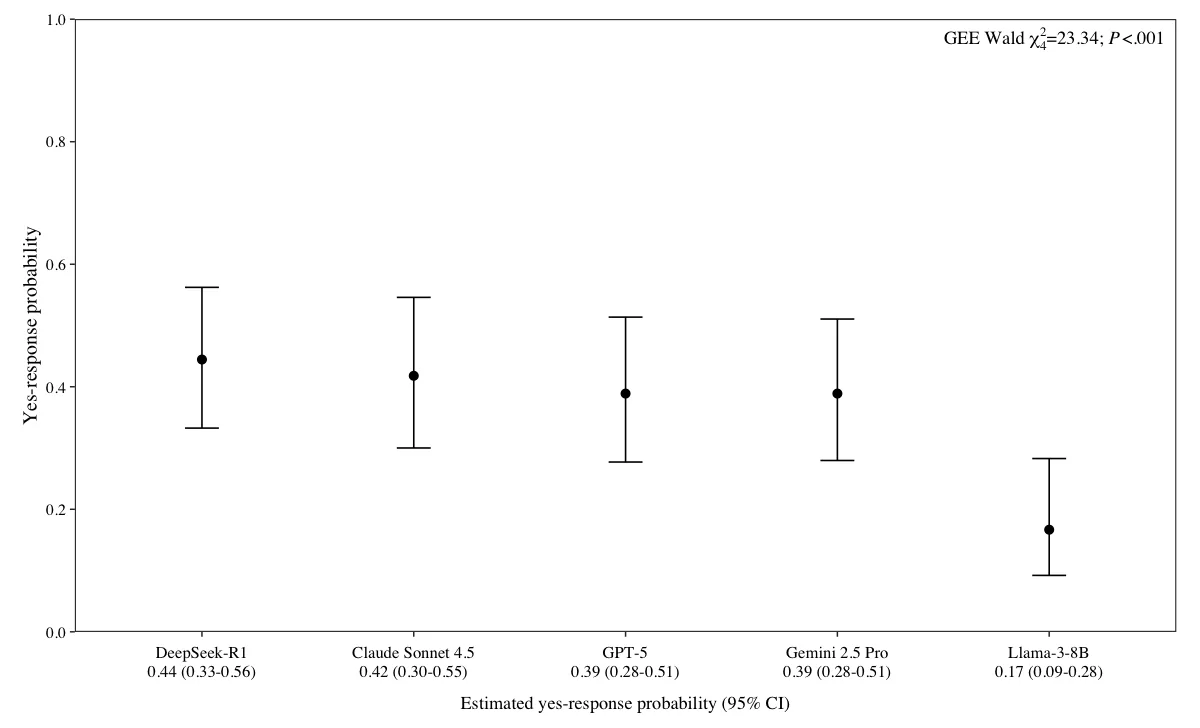
Model-estimated probabilities of a yes response across the 5 primary large language models (LLMs). Estimates are from the trial-level case-clustered generalized estimating equation (GEE); error bars indicate 95% CIs.

**Table 3:** Primary model results (N=180)^a^.

Models	Valid outputs^b^ (N=180), n	Yes responses^c^, n/N (%)	Case-majority yes^d^, n/N (%)	Within-model response stability across 3 runs^e^, n/N (%)	Confidence^f^, mean (SD)	Confidence, median (IQR)
DeepSeek-R1	180	80/180 (44.4)	27/60 (45)	51/60 (85)	0.85 (0.08)	0.85 (0.80-0.95)
Claude Sonnet 4.5^g^	177	75/177 (42.4)	25/59 (42.4)	59/59 (100)	0.77 (0.12)	0.75 (0.65-0.92)
GPT-5	180	70/180 (38.9)	23/60 (38.3)	57/60 (95)	0.81 (0.11)	0.82 (0.74-0.90)
Gemini 2.5 Pro	180	70/180 (38.9)	24/60 (40)	54/60 (90)	0.90 (0.14)	0.95 (0.85-0.98)
Llama-3-8B	180	30/180 (16.7)	10/60 (16.7)	60/60 (100)	0.83 (0.05)	0.80 (0.80-0.90)

^a^Trial-level binary comparisons used a case-clustered generalized estimating equation; the overall model effect was Wald *χ*^2^_4_=23.34 (*P*<.001).

^b^Valid outputs are reported relative to the 180 planned trial-level outputs for each model.

^c^Yes-response percentages use valid trial-level binary answers as the denominator.

^d^Case-majority yes uses cases with a valid majority answer across the 3 runs.

^e^Within-model response stability is the proportion of cases with identical valid binary answers across all 3 runs.

^f^Confidence denotes the model-reported self_confidence for the selected answer and is not a calibrated probability or a probability of ethical correctness.

^g^Claude Sonnet 4.5 refused case 44 in all 3 trials; the refusals were not coded as no.

### Pairwise Comparisons of Positive Response Rates

After Holm adjustment, DeepSeek-R1 (OR 4.00, 95% CI 2.02-7.90; adjusted *P*<.001), Claude Sonnet 4.5 (OR 3.59, 95% CI 1.87-6.88; adjusted *P*=.001), GPT-5 (OR 3.18, 95% CI 1.55-6.53; adjusted *P*=.011), and Gemini 2.5 Pro (OR 3.18, 95% CI 1.56-6.47; adjusted *P*=.011) each had higher odds of a yes response than Llama-3-8B. No other pairwise comparison was significant after adjustment (Figure 2).

**Figure 2:**
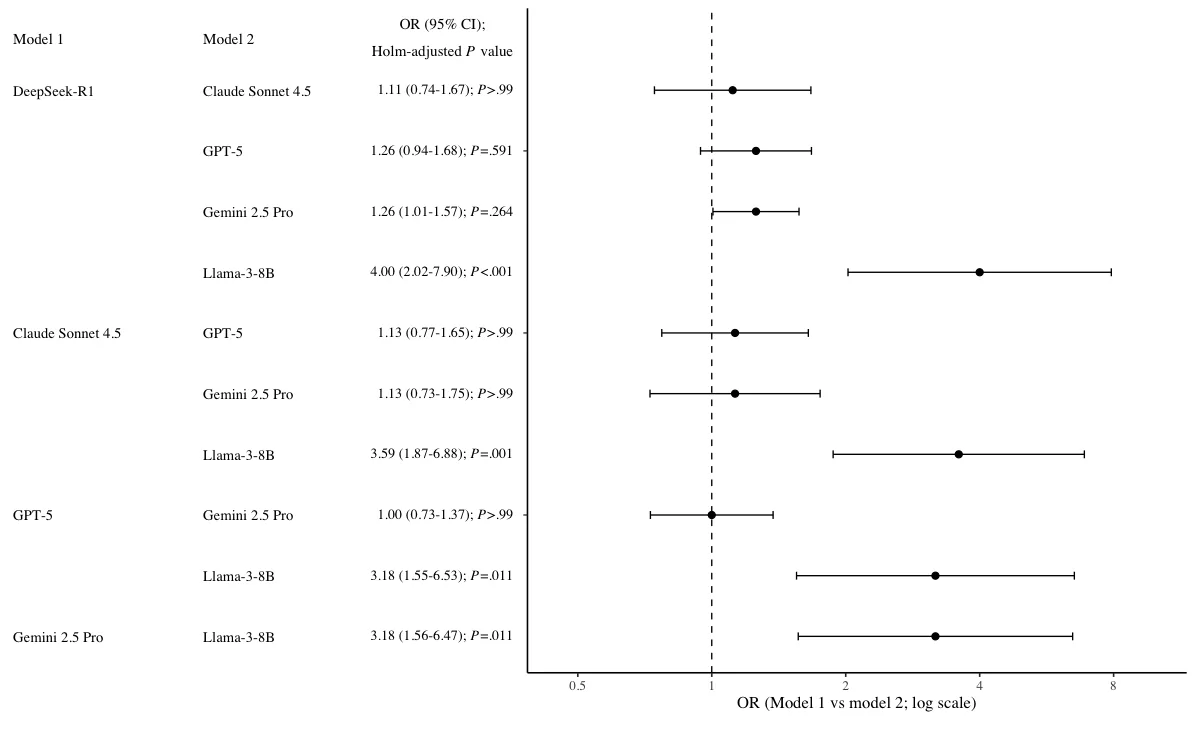
Generalized estimating equation (GEE)-estimated odds ratios (ORs) for yes responses, accounting for within-case correlation. ORs are shown on a logarithmic scale with 95% CIs; Holm adjustment was applied to *P* values only. ORs greater than 1 favor model 1 in each comparison.

### Model-Reported Confidence

Median model-reported confidence was 0.75 (IQR 0.65-0.92) for Claude Sonnet 4.5, 0.85 (IQR 0.80-0.95) for DeepSeek-R1, 0.95 (IQR 0.85-0.98) for Gemini 2.5 Pro, 0.82 (IQR 0.74-0.90) for GPT-5, and 0.80 (IQR 0.80-0.90) for Llama-3-8B.

### Within-Model Stability

Within-model response stability across 3 runs was 95% (57/60) for GPT-5, 100% (59/59) for Claude Sonnet 4.5 among cases with valid binary outputs, 90% (54/60) for Gemini 2.5 Pro, 85% (51/60) for DeepSeek-R1, and 100% (60/60) for Llama-3-8B. Pooled raw agreement across the 3 run pairs within each model ranged from 90.0% (162/180 comparisons) to 100.0% (177/177 and 180/180 comparisons), and run-level Fleiss κ ranged from 0.80 to 1.00.

**Agreement Statistics**

Across the 59 complete-panel cases, raw agreement was 79.0% (466/590 model-pair comparisons; 95% bootstrap CI 72.9%-84.7%), and Fleiss κ was 0.55 (95% bootstrap CI 0.43-0.66). Pairwise Cohen κ ranged from 0.23 to 0.83 (Figure 3; Table S3 in Multimedia Appendix 1). Overall and exploratory subgroup estimates are reported in Figure 4 and Table 4.

**Figure 3:**
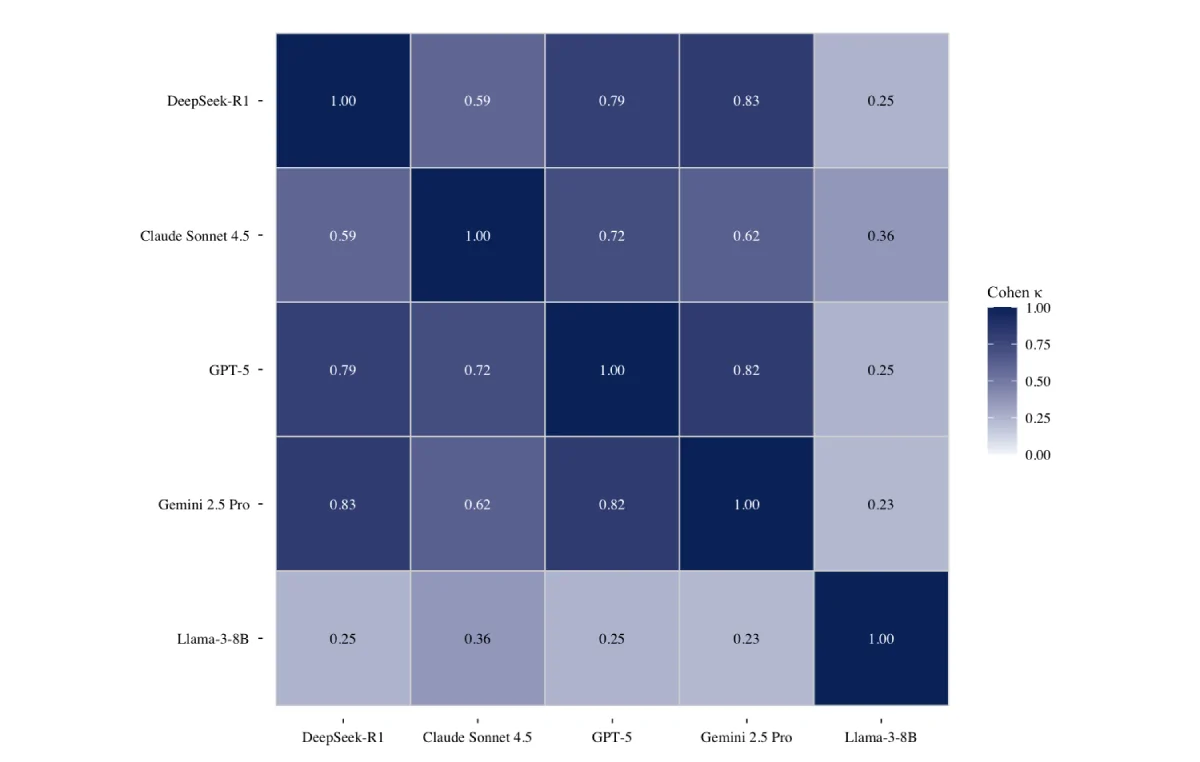
Pairwise agreement among the 5 primary large language models (LLMs) across 59 complete-panel cases. The heatmap displays Cohen κ for each model pair; pairwise raw agreement and 95% CIs are provided in Table S3 in Multimedia Appendix 1.

**Figure 4:**
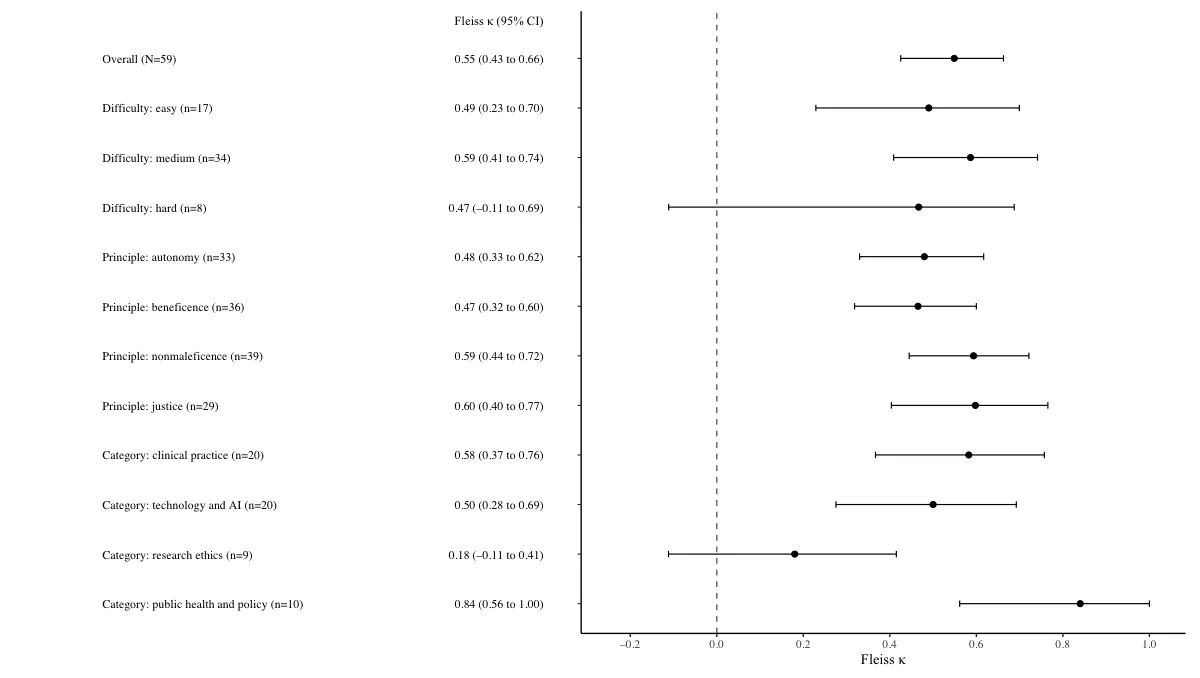
Overall and exploratory subgroup intermodel agreement. Fleiss κ is shown for the complete panel and by difficulty, ethical principle, and clinical category; error bars indicate bootstrap 95% CIs.

**Table 4:** Agreement and discordance by subgroup^a^.

Panels and groups			Raw agreement (%), score (95% CI)		Fleiss κ (95% CI)		Any discordance, n (%)		4:1 discordance, n (%)
Overall (n=59)			79.0 (72.9-84.7)		0.55 (0.43 to 0.66)		27 (45.8)		19 (32.2)
**Difficulty**									
	Easy (n=17)	75.3 (63.5-87.1)		0.49 (0.23 to 0.70)		9 (52.9)		6 (35.3)	
	Medium (n=34)	80.6 (72.4-88.2)		0.59 (0.41 to 0.74)		14 (41.2)		9 (26.5)	
	Hard (n=8)	80.0 (65.0-95.0)		0.47 (−0.11 to 0.69)		4 (50)		4 (50)	
**Ethical principle**									
	Autonomy (n=33)	75.8 (67.9-83.6)		0.48 (0.33 to 0.62)		18 (54.5)		14 (42.4)	
	Beneficence (n=36)	75.0 (67.2-82.2)		0.47 (0.32 to 0.60)		20 (55.6)		15 (41.7)	
	Nonmaleficence (n=39)	81.5 (74.4-88.7)		0.59 (0.44 to 0.72)		16 (41)		12 (30.8)	
	Justice (n=29)	80.7 (71.7-89.0)		0.60 (0.40 to 0.77)		12 (41.4)		8 (27.6)	
**Category**									
	Clinical practice (n=20)	81.0 (72.0-90.0)		0.58 (0.37 to 0.76)		9 (45)		8 (40)	
	Technology and AI (n=20)	79.0 (68.0-89.0)		0.50 (0.28 to 0.69)		9 (45)		6 (30)	
	Research ethics (n=9)	60.0 (46.7-75.6)		0.18 (−0.11 to 0.41)		7 (77.8)		3 (33.3)	
	Public health and policy (n=10)	92.0 (80.0-100.0)		0.84 (0.56 to 1.00)		2 (20)		2 (20)	

^a^Analyses used the 59 cases with valid case-majority answers from all 5 primary models. The n in subgroup labels is the number of eligible cases; in the discordance columns, n is the number of discordant cases. Any discordance includes both 4:1 and 3:2 splits. CIs are percentile intervals from 10,000 case-level bootstrap samples. Ethical principle groups overlap. All subgroup analyses were exploratory.

### Exploratory Subgroup Analyses

Exploratory agreement was lowest for research ethics (n=9; raw agreement 60.0%, 54/90 model-pair comparisons, 95% bootstrap CI 46.7%-75.6%; Fleiss κ=0.18, 95% bootstrap CI −0.11 to 0.41). For hard cases, raw agreement was 80.0% (64/80 model-pair comparisons; 95% bootstrap CI 65.0%-95.0%), and Fleiss κ was 0.47 (95% bootstrap CI −0.11 to 0.69; n=8).

### Discordance Analysis

Among 59 complete-panel cases, any between-model discordance occurred in 27 (45.8%), including 19 (32.2%) with a 4:1 split and 8 (13.6%) with a 3:2 split (Table 4). Among the 19 cases with a 4:1 split, Llama-3-8B was the sole discordant model in 13 cases (12 no cases and 1 yes case), Claude Sonnet 4.5 in 3 cases (all yes), DeepSeek-R1 in 2 cases (both yes), and Gemini 2.5 Pro in 1 case (yes); GPT-5 was not the sole discordant model in any 4:1 case (Table 5).

**Table 5:** Composition of the 4:1 cases (N=19)^a^.

Unique model in 4:1 split	Unique answer	Cases, n (%)
Claude Sonnet 4.5	Yes	3 (15.8)
DeepSeek-R1	Yes	2 (10.5)
Gemini 2.5 Pro	Yes	1 (5.3)
Llama-3-8B	No	12 (63.2)
Llama-3-8B	Yes	1 (5.3)

^a^This table describes the composition of the 19 cases with a 4:1 split and should not be interpreted as a subgroup-specific probability of discordance. No 4:1 split had GPT-5 as the unique model.

### Reason Analyses

The complete results of the answer-reason directional consistency audit and cross-model reason review are presented in Tables S4 and S5 in Multimedia Appendix 1.

### Annotation Agreement

The complete preconsensus agreement results for the difficulty and ethical principle annotations are presented in Table S6 in Multimedia Appendix 1.

### Exploratory Small-Model Analysis

In the exploratory analysis, DeepSeek-R1-Distill-Qwen-1.5B returned 114 yes responses out of 180 outputs (63.3%). All other results for this model are reported in Table S1 in Multimedia Appendix 1.

## Discussion

### Principal Findings

We aimed to compare recommendation direction, model-reported confidence, within-model stability, and between-model agreement across selected contemporary LLMs using a standardized set of medical ethics cases. Across the 5-model primary panel, trial-level yes rates ranged from 16.7% (30/180) for Llama-3-8B-Instruct to 44.4% (80/180) for full-size DeepSeek-R1. The overall model effect was significant, but after Holm adjustment, only comparisons involving Llama-3-8B were significant. Across 59 complete-panel cases, raw agreement was 79.0% (466/590 model-pair comparisons; Fleiss κ=0.55), and within-model response stability across 3 runs ranged from 85.0% (51/60) to 100.0% (59/59 and 60/60).

### Comparison With Prior Work

Prior studies have generally evaluated accuracy against reference answers. Ethics of AI in Medical Contexts (ETHICS) measures prediction of labeled everyday moral judgments [7]; medical studies have used multiple-choice bioethics or USMLE-style ethics and soft skill questions [8-10]; and Massive Multitask Language Understanding (MMLU) measures multitask accuracy across academic and professional subjects, including morality and medicine [20]. Our cases intentionally lack normative reference answers; therefore, our end points characterize recommendation variation, confidence, and stability rather than accuracy. Medical ethics often requires weighing competing principles and contextual values rather than identifying a single verifiable ground truth [4,21]. Balas et al [22] asked 6 ethicists to assess GPT-4 responses to 8 complex vignettes for relevance, reasoning, depth, clarity, and acceptability and reported limitations in nuanced, context-sensitive reasoning. MedEthicEval assessed both medical ethics knowledge and application across scenarios ranging from clear violations to dilemmas without obvious resolutions [23]. Our study complements these approaches by applying the same cases repeatedly across 5 model families and quantifying recommendation direction, model-reported confidence, within-model stability, and between-model variation. These end points do not determine ethical correctness. Instead, this study provides a standardized, version-specific framework for auditing recommendation tendencies and repeatability across models, complementing rather than replacing expert ethics assessment [24].

### Interpretation and Implications

This audit set compares recommendation direction, confidence, and stability; it does not assess ethical correctness or fairness. Although demographic attributes were minimized, this did not create a validated neutral control group. These cases provide structured templates for future counterfactual studies using prespecified matched pairs that differ only in 1 sensitive attribute and corresponding paired analyses [25]. Model-reported confidence supplemented the binary answer but was not interpreted as a calibrated probability or as evidence of ethical correctness. The exploratory DeepSeek-R1-Distill-Qwen-1.5B model had a higher yes rate than the primary models (63.3% [114/180] vs 16.7% [30/180] to 44.4% [80/180]), but this single comparison cannot identify the effects of model size, distillation, or instruction following. Principal justifications differed materially in 2 of the 7 sampled unanimous-no cases, showing that binary agreement did not always imply equivalent reasoning. This 14-case review does not estimate the prevalence of divergent reasoning or support model-level philosophical conclusions. Because model scale, architecture, training, alignment, infrastructure, and settings varied together, this study cannot identify why models differed. Associations with distillation [18] or constitutional AI [26] are therefore hypotheses, not causal explanations. Automation bias has been documented in clinical decision support settings [27,28]. The observed model-dependent recommendation tendencies provide testable hypotheses for prospective studies examining clinician reliance, decisions to act or withhold action, and downstream outcomes. The current endorsement and refusal rates do not establish these clinical effects. Until prospective evidence is available, human review and institutional governance remain important when LLM outputs are considered in ethically sensitive settings.

### Limitations

Our study has several limitations inherent to the evolving nature of LLM evaluation. First, our assessment relied on a standardized zero-shot prompting strategy to ensure strict comparability across architectures. However, LLM performance can be sensitive to subtle changes in prompt formatting [29]. The study was conducted only in English. Ethical framing is culturally situated, and language may interact with the data and alignment processes used to develop each model. However, we did not measure Western-centric bias and cannot infer it from the use of English alone. Multilingual and cross-cultural paired evaluations are needed before generalizing the findings to other linguistic or bioethical contexts [30]. Subgroup analyses were exploratory, and small groups produced wide CIs; subgroup rankings should not be interpreted as stable differences. The concise scenarios (median 18, IQR 16-21 words) isolate a single decision axis but omit clinical context, stakeholder relationships, institutional constraints, and evolving uncertainty. They therefore do not reproduce the complexity of real clinical ethics consultation. The panel did not include a formally trained bioethicist or clinical ethicist, and R1 both drafted and annotated the cases. The rubric and agreement analysis support transparency and internal reproducibility but do not establish content or criterion validity. External validation should include independent ethics expertise and multiple cultural settings. Three runs characterize short-term stability but do not remove version or time dependence, and the observational comparison cannot isolate model design factors. Unrecorded generation settings may still limit exact replication. Results therefore apply to the documented versions and query period [31].

### Conclusions

Selected contemporary LLMs showed model-dependent variation in binary recommendations for the same medical ethics cases, despite generally high short-term within-model stability, while overall between-model agreement was moderate. These findings support transparent reporting of model versions, recommendation tendencies, confidence, and repeatability when LLMs are evaluated for ethically sensitive medical applications [32]. Because behavioral agreement does not establish ethical correctness or clinical benefit, model evaluation should be complemented by human ethical review and external validation.

## Appendix

Multimedia Appendix 1 Case annotation procedures, answer-reason audits, model execution information, and exploratory agreement results.

## Abbreviations

CSV: comma-separated values
DFKI: German Research Center for Artificial Intelligence
ETHICS: Ethics of AI in Medical Contexts
GEE: generalized estimating equation
LLM: large language model
MMLU: Massive Multitask Language Understanding
OR: odds ratio
R1: reviewer 1
R2: reviewer 2
R3: reviewer 3
TRIPOD: Transparent Reporting of a Multivariable Prediction Model for Individual Prognosis or Diagnosis
USMLE: United States Medical Licensing Examination

## Data Availability

The dataset generated and analyzed during this study is available in the Hugging Face datasets repository [33]. Analysis-ready derived data and code for the primary statistical analyses and generation of the main figure and tables are available in the Hugging Face dataset [33]. The repository includes reproducibility instructions, software versions, data dictionaries, and licensing information.
