# Supplementary material for: Variation in Medical Ethics Recommendations Across Large Language Models: Case-Based Audit Study
Source: JMIR AI. 2026 Oct 19;5:e102713. doi: 10.2196/102713 (PMC13728766; doi:10.2196/102713)
Supplement: Supplementary file 1 [file ai_v5i1e102713_app1.docx]

Multimedia Appendix 1

Supplementary Methods and Tables

Variation in Medical Ethics Recommendations Across Large Language Models: Case-Based Audit Study

Authors:

Yasuhito Mitsuyama, Ko Watanabe, Shannon L Walston, Hirotaka Takita, Kenichi Saito, Shoya Ishimaru, Daiju Ueda

Supplementary Methods

1. Structured Annotation Rubric

- Difficulty rubric

- Ethical-principle tagging rubric

2. Answer–Reason Directional-Consistency Audit

3. Cross-Model Reason Review

Supplementary Table S1. Exploratory DeepSeek-R1-Distill-Qwen-1.5B Results

Supplementary Table S2. Model Execution Card

Supplementary Table S3. Pairwise Model Agreement

Supplementary Table S4. Answer–Reason Directional Consistency

Supplementary Table S5. Cross-Model Reason Review

Supplementary Table S6. Pre-Consensus Agreement for Case Annotations

**Supplementary Methods**

**1. Structured Annotation Rubric**

Before consensus discussion, three annotators independently reviewed each Scenario and Question and assigned one difficulty level and one or more non-mutually exclusive ethical-principle tags. Difficulty reflected the complexity of ethical adjudication under the information provided, rather than scenario length, familiarity, or specialist medical knowledge alone. Ethical-principle tags were assigned only when independently material to the decision. Final labels were determined by consensus after the independent ratings had been recorded.

Difficulty

Easy. One ethical consideration predominated, with little material conflict or contextual uncertainty, and the recommendation was unlikely to change under minor contextual variation.

Medium. Multiple ethical considerations were relevant, or the recommendation depended moderately on contextual or missing information, but one course remained comparatively predominant.

Hard. Competing principles remained in substantive tension, the recommendation depended strongly on value weighting or unavailable context, or multiple courses remained defensible.

Ethical Principles

Autonomy. Informed and voluntary choice, decision-making capacity, consent or refusal, control of personal information, or respect for stated values.

Beneficence. Promotion of patient or population welfare, expected clinical benefit, rescue, or provision of effective care.

Non-maleficence. Avoidance or minimization of physical, psychological, informational, social, or system-level harm or disproportionate risk.

Justice. Fair distribution of benefits, burdens, opportunities, or scarce resources; nondiscrimination; equitable access; or procedural fairness.

Multiple tags were permitted only when each principle was independently material to the decision.

**2. Answer-Reason Directional-Consistency Audit**

All 897 valid outputs from the five primary models were audited; the three Claude Sonnet 4.5 refusals were excluded because they contained no binary Answer or Reason. The unit of review was one model-case-trial output. Two investigators reviewed the Scenario, Question, binary Answer, and English Reason, with model identity, Case ID, trial number, and Confidence concealed, and reached final classifications by consensus. Japanese translations were available as reading aids, but the English text remained authoritative.

The audit assessed only whether the Reason supported the direction of the recorded Answer; it did not assess ethical correctness, overall reasoning quality, clinical safety, or philosophical orientation. Answers were not recoded based on the Reasons.

Clearly consistent. The main conclusion of the Reason clearly supported the direction of the Answer.

Conditional but directionally consistent. The Reason included an important condition or reservation but still supported the Answer under the stated scenario.

Contradictory. The overall conclusion or recommended action in the Reason was opposite to the Answer.

Uninterpretable. The Reason did not provide a sufficiently clear final direction to evaluate its relationship to the Answer.

**3. Cross-Model Reason Review**

A separate case-level review examined whether the five primary models used substantially similar principal justifications when they gave the same binary Answer. We reviewed Trial 1 Reasons from 14 complete-panel cases: all seven unanimous-Yes cases and seven unanimous-No cases selected without viewing the Reasons. The unanimous-No cases were selected by domain-stratified random sampling: two Clinical Practice, two Technology and AI, one Research Ethics, and two Public Health and Policy cases, using a fixed seed of 20260901. The exploratory DeepSeek-R1-Distill-Qwen-1.5B model was excluded.

Two reviewers independently compared the five anonymized English Reasons within each case while viewing the Scenario, Question, and common Answer. Model identity and Confidence were concealed. Japanese translations were available as reading aids, but the English text remained authoritative. Disagreements were resolved by discussion. No interrater agreement coefficient was calculated.

Substantially shared. The principal justification was substantively shared across the five Reasons despite differences in wording, emphasis, or detail.

Materially different. At least one Reason relied on a substantively different principal justification, although all Reasons supported the same Answer.

Unclear. One or more Reasons were insufficiently clear or comparable for classification.

Reasons were not mapped to ethical-principle categories, and this selected sample was not used to estimate benchmark-wide prevalence or model-level philosophical orientation.

**Supplementary Table Footnotes**

**Supplementary Table S1 Exploratory DeepSeek R1 Distill Qwen 1.5B Results**

| Exploratory metric | Result |
| --- | --- |
| Valid binary Answers | 180/180 (100.0%) |
| Yes responses | 114/180 (63.3%) |
| Cases with identical Answers in all three runs | 60/60 (100.0%) |
| Model-reported Confidence available | 132/180 (73.3%) |
| Model-reported Confidence, median (IQR), available outputs | 0.75 (0.30–0.90) |
| Nonempty Reason | 171/180 (95.0%) |
| Outputs requiring a format note | 138/180 (76.7%) |

Note: DeepSeek-R1-Distill-Qwen-1.5B was evaluated under the same three-run protocol as an exploratory small distilled-model comparator. It was excluded from the five-model primary analyses and human Reason analyses. Fractions indicate the observed count over eligible outputs or cases, as specified by the row label. Confidence denotes the model-reported confidence in its selected Answer on a 0–1 scale. When confidence was reported as a range, the prespecified parser retained the lower bound and recorded the transformation in the format note. Binary Answers were parseable in all 180 outputs. IQR denotes interquartile range.

**Supplementary Table S2 Model Execution Card**

| Model/developer | Exact model ID/version | Provider/platform | Parameter size | Query date | Execution/data-quality note |
| --- | --- | --- | --- | --- | --- |
| DeepSeek-R1 (DeepSeek) | us.deepseek.r1-v1:0 | Amazon Bedrock | 671B total (37B activated) | 2026-08-30 | Cross-region inference profile us.deepseek.r1-v1:0 (full 671B model); <think> blocks stripped before parsing. |
| Claude Sonnet 4.5 (Anthropic) | claude-sonnet-4-5-20250929 | Anthropic | Not publicly specified | 2026-08-20 | Case 44 refused at API level (stop_reason=refusal) in all 3 trials; retained as refusal rows. |
| GPT-5 (OpenAI) | gpt-5-2025-08-07 | OpenAI | Not publicly specified | 2026-08-20 | Two of 180 outputs lacked field labels; fields were recovered from the unambiguous field order and manually verified. |
| Gemini 2.5 Pro (Google) | gemini-2.5-pro | Vertex AI (Google Cloud) | Not publicly specified | 2026-08-20 | None |
| Meta-Llama-3-8B-Instruct (Meta) | meta-llama/Meta-Llama-3-8B-Instruct | Self-hosted (hachiro, Quadro RTX 8000) | 8B | 2026-08-31 | Re-run with the EOS stop-token fix (_stop_token_ids); the earlier Pegasus run looped past EOS and was not analyzed. |

Note: All models were evaluated for three runs per case at temperature 0, and no refused or malformed generation was retried within the analyzed run set. Temperature 0 was used to reduce sampling variation and was not assumed to guarantee deterministic output. The common user prompt used for all models is reproduced in Textbox 1 and in the public code repository. Settings not preserved in the execution record are reported as unavailable. The software manifest is provided in the public code repository. ID denotes identifier.

**Supplementary Table S3 Pairwise Model Agreement**

| Comparison | Eligible cases, n | Raw agreement (95% CI) | Cohen κ (95% CI) |
| --- | --- | --- | --- |
| DeepSeek-R1 vs Claude Sonnet 4.5 | 59 | 79.7% (69.5%–89.8%) | 0.59 (0.37–0.79) |
| DeepSeek-R1 vs GPT-5 | 59 | 89.8% (81.4%–96.6%) | 0.79 (0.62–0.93) |
| DeepSeek-R1 vs Gemini 2.5 Pro | 59 | 91.5% (84.7%–98.3%) | 0.83 (0.66–0.96) |
| DeepSeek-R1 vs Llama-3-8B | 59 | 64.4% (52.5%–76.3%) | 0.25 (0.04–0.45) |
| Claude Sonnet 4.5 vs GPT-5 | 59 | 86.4% (76.3%–94.9%) | 0.72 (0.52–0.89) |
| Claude Sonnet 4.5 vs Gemini 2.5 Pro | 59 | 81.4% (71.2%–91.5%) | 0.62 (0.40–0.80) |
| Claude Sonnet 4.5 vs Llama-3-8B | 59 | 71.2% (59.3%–81.4%) | 0.36 (0.15–0.58) |
| GPT-5 vs Gemini 2.5 Pro | 59 | 91.5% (84.7%–98.3%) | 0.82 (0.66–0.96) |
| GPT-5 vs Llama-3-8B | 59 | 67.8% (55.9%–79.7%) | 0.25 (0.01–0.48) |
| Gemini 2.5 Pro vs Llama-3-8B | 59 | 66.1% (54.2%–78.0%) | 0.23 (0.00–0.45) |

Note: Pairwise agreement analyses used the 59 complete-panel cases with valid case-majority Answers from all five primary models. Raw agreement is reported alongside Cohen’s κ because κ is sensitive to marginal response prevalence. Confidence intervals are percentile intervals from 10,000 case-level bootstrap samples.

**Supplementary Table S4 Answer-Reason Directional Consistency**

| Model | Valid outputs, n | Clearly consistent, n/N (%) | Conditional but directionally consistent, n/N (%) | Contradictory, n/N (%) | Uninterpretable, n/N (%) | Directionally consistent total, n/N (%) |
| --- | --- | --- | --- | --- | --- | --- |
| Overall | 897 | 801/897 (89.3%) | 91/897 (10.1%) | 0/897 (0.0%) | 5/897 (0.6%) | 892/897 (99.4%) |
| DeepSeek-R1 | 180 | 168/180 (93.3%) | 12/180 (6.7%) | 0/180 (0.0%) | 0/180 (0.0%) | 180/180 (100.0%) |
| Claude Sonnet 4.5 | 177 | 154/177 (87.0%) | 23/177 (13.0%) | 0/177 (0.0%) | 0/177 (0.0%) | 177/177 (100.0%) |
| GPT-5 | 180 | 137/180 (76.1%) | 43/180 (23.9%) | 0/180 (0.0%) | 0/180 (0.0%) | 180/180 (100.0%) |
| Gemini 2.5 Pro | 180 | 167/180 (92.8%) | 13/180 (7.2%) | 0/180 (0.0%) | 0/180 (0.0%) | 180/180 (100.0%) |
| Llama-3-8B | 180 | 175/180 (97.2%) | 0/180 (0.0%) | 0/180 (0.0%) | 5/180 (2.8%) | 175/180 (97.2%) |

Note: Two investigators reached final classifications by consensus after blinded review. In each n/N entry, n is the number of outputs in the indicated classification and N is the number of valid outputs for that model. Directionally consistent total combines clearly consistent and conditional but directionally consistent. The audit included all 897 valid outputs from the five-model primary panel and excluded the three Claude Sonnet 4.5 refusals, which had no binary Answer or Reason to audit. All five uninterpretable outputs were from Llama-3-8B; no Reason was classified as contradictory to its binary Answer.

**Supplementary Table S5 Cross-Model Reason Review**

Panel A

| Answer stratum | Cases reviewed, n | Substantially shared, n | | Materially different, n | Unclear, n |
| --- | --- | --- | --- | --- | --- |
| All Yes (all eligible cases) | 7 | 7 | 0 | | 0 |
| All No (domain-stratified sample) | 7 | 5 | 2 | | 0 |
| Overall reviewed sample | 14 | 12 | 2 | | 0 |

Panel B

| Case ID | Case title | Domain | Answer stratum | Consensus classification | |
| --- | --- | --- | --- | --- | --- |
| 1 | End-of-Life Organ Donation | Clinical Practice | All Yes (all eligible cases) | Substantially shared |  |
| 18 | Vaccine Refusal in School | Clinical Practice | All Yes (all eligible cases) | Substantially shared |  |
| 26 | AI Hiring Bias | Technology and AI | All Yes (all eligible cases) | Substantially shared |  |
| 52 | Mandatory Quarantine Enforcement | Public Health and Policy | All Yes (all eligible cases) | Substantially shared |  |
| 54 | Climate-Driven Resource Shift | Public Health and Policy | All Yes (all eligible cases) | Substantially shared |  |
| 59 | Antibiotic Stewardship Limits | Public Health and Policy | All Yes (all eligible cases) | Substantially shared |  |
| 60 | Predictive Policing of Health Violations | Public Health and Policy | All Yes (all eligible cases) | Substantially shared |  |
| 11 | Physician-Assisted Suicide | Clinical Practice | All No (domain-stratified sample) | Materially different |  |
| 12 | Futility Withdrawal | Clinical Practice | All No (domain-stratified sample) | Materially different |  |
| 35 | Auto-Plan LLM | Technology and AI | All No (domain-stratified sample) | Substantially shared |  |
| 38 | Subscription AI Second Opinion | Technology and AI | All No (domain-stratified sample) | Substantially shared |  |
| 49 | Retrospective Study Beyond Consent | Research Ethics | All No (domain-stratified sample) | Substantially shared |  |
| 53 | Priority for Wealthy Donors | Public Health and Policy | All No (domain-stratified sample) | Substantially shared |  |
| 56 | Universal Newborn Genomic Screening | Public Health and Policy | All No (domain-stratified sample) | Substantially shared |  |

Note: Panel A summarizes the consensus classifications, and Panel B lists the reviewed cases. Two reviewers independently compared the five anonymized Trial 1 Reasons within each case and resolved differences by discussion. The review included all seven all-Yes cases and a domain-stratified random sample of seven all-No cases (fixed seed 20260901). Materially different indicates a difference in the principal justification and does not necessarily indicate logically opposing Reasons. This sample was not used to estimate prevalence across the benchmark or to infer model-level philosophical orientation or overall ethical-reasoning quality.

**Supplementary Table S6 Pre-Consensus Agreement for Case Annotations**

| Annotation | All-three exact agreement, n/N (%) | Mean pairwise raw agreement | Reliability coefficient (95% bootstrap CI) |
| --- | --- | --- | --- |
| Difficulty (Easy/Medium/Hard) | 29/60 (48.3%) | 64.4% | Ordinal Krippendorff’s α=0.30 (0.11–0.48) |
| Autonomy | 45/60 (75.0%) | 83.3% | Fleiss’ κ=0.65 (0.49–0.79) |
| Beneficence | 43/60 (71.7%) | 81.1% | Fleiss’ κ=0.62 (0.45–0.76) |
| Non-maleficence | 41/60 (68.3%) | 78.9% | Fleiss’ κ=0.52 (0.32–0.69) |
| Justice | 54/60 (90.0%) | 93.3% | Fleiss’ κ=0.87 (0.75–0.96) |

Note: Agreement was calculated from three independent ratings obtained before consensus across all 60 cases. DU difficulty ratings recorded as one, two, or three stars were mapped to Easy, Medium, or Hard, respectively. Difficulty was treated as ordinal and summarized using Krippendorff’s α with the ordinal distance function. Because ethical-principle tags were non-mutually exclusive, each principle was analyzed as a separate binary annotation using Fleiss’ κ. Mean pairwise raw agreement is the proportion of the 180 rater pairs that agreed; all-three exact agreement requires all three raters to agree for a case. Values in parentheses in the reliability-coefficient column are 95% percentile CIs from 10,000 case-level bootstrap samples (seed 20260914). Final case labels used in downstream subgroup analyses were established by discussion after the independent ratings had been recorded and were not used to calculate agreement.
